# Potential risk factors associated with human alveolar echinococcosis: Systematic review and meta-analysis

**DOI:** 10.1371/journal.pntd.0005801

**Published:** 2017-07-17

**Authors:** Franz J. Conraths, Carolina Probst, Alessia Possenti, Belgees Boufana, Rosella Saulle, Giuseppe La Torre, Luca Busani, Adriano Casulli

**Affiliations:** 1 Friedrich-Loeffler-Institut, Institute of Epidemiology, Greifswald-Insel Riems, Germany; 2 Department of Infectious Diseases, Istituto Superiore di Sanità (ISS), Rome, Italy; 3 European Union Reference Laboratory for Parasites (EURLP), ISS, Rome, Italy; 4 World Health Organization Collaborating Centre for the epidemiology, detection and control of cystic and alveolar echinococcosis (in humans and animals), ISS, Rome, Italy; 5 Sapienza University of Rome, Department of Public Health and Infectious Diseases, Rome, Italy; 6 Department of Food Safety and Veterinary Public Health, Istituto Superiore di Sanità (ISS), Rome, Italy; University of Zurich, SWITZERLAND

## Abstract

**Background:**

Human alveolar echinococcosis (AE) is a severe zoonotic disease caused by the metacestode stage of *Echinococcus multilocularis*. AE is commonly associated with a long incubation period that may last for more than ten years. The objective of this systematic literature review was to identify and summarize the current knowledge on statistically relevant potential risk factors (PRFs) associated with AE in humans.

**Methodology/Principal findings:**

Six bibliographic databases were searched, generating a total of 1,009 publications. Following the removal of duplicate records and the exclusion of papers that failed to meet the criteria of a previously agreed *a priori* protocol, 23 publications were retained; however, 6 of these did not contain data in a format that allowed their inclusion in the meta-analysis. The remaining 17 publications (6 case-control and 11 cross-sectional studies) were meta-analysed to investigate associations between AE and PRFs. Pooled odds ratios (OR) were used as a measure of effect and separately analysed for case-control and cross-sectional studies. In the case-control studies, the following PRFs for human AE showed higher odds of outcome: “dog ownership”, “cat ownership”, “have a kitchen garden”, “occupation: farmer”, “haymaking in meadows not adjacent to water”, “went to forests for vocational reasons”, “chewed grass” and “hunting / handling foxes”. In the cross-sectional studies, the following PRFs showed higher odds of outcome: “dog ownership”, “play with dogs”, “gender: female”, “age over 20 years”, “ethnic group: Tibetan”, “low income”, “source of drinking water other than well or tap”, “occupation: herding” and “low education”. Our meta-analysis confirmed that the chance of AE transmission through ingestion of food and water contaminated with *E*. *multilocularis* eggs exists, but showed also that food- and water-borne PRFs do not significantly increase the risk of infection.

**Conclusions/significance:**

This systematic review analysed international peer-reviewed articles that have over the years contributed to our current understanding of the epidemiology of human AE. The identification of potential risk factors may help researchers and decision makers improve surveillance and/or preventive measures that aim at decreasing human infection with *E*. *multilocularis*. More primary studies are needed to confirm potential risk factors and their role in the epidemiology of human AE.

## Author summary

Human alveolar echinococcosis is a severe zoonotic disease caused by the metacestode stage of the tapeworm *Echinococcus multilocularis*. The objective of this systematic literature review was to identify and summarize the current knowledge on potential risk factors associated with human alveolar echinococcosis. The categories of potential risk factors included dog-related factors such as dog ownership or play with dogs; vocational factors like being a farmer or handling foxes; human habits such as chewing grass; gender (being female) and socio-cultural factors like being Tibetan or having a low income or poor education, which may be relevant only in particular endemic areas. The identification of potential risk factors may help identify strategies that aim to decrease human infection with *E*. *multilocularis*.

## Introduction

Alveolar echinococcosis (AE) is considered as one of the most dangerous parasitic zoonoses occurring in the northern hemisphere. The life cycle of the parasite is maintained by definitive hosts shedding eggs with the faeces and the subsequent ingestion of these eggs by suitable intermediate hosts. Definitive hosts become infected by eating intermediate hosts, in which the metacestode stage develops. Carnivores, in particular the red fox in Europe and the raccoon dog among wildlife, as well as the dog and to a lesser extent the cat, represent definitive hosts for the parasite [1–4]. In other epidemiological settings, for example in the People's Republic of China, dogs may, in addition to foxes, play an important role in the transmission of AE [5]. Small rodents, mainly voles, serve as natural intermediate hosts.

Humans are aberrant intermediate hosts and are affected by the metacestode stage of the parasite. They become infected through the ingestion of *E*. *multilocularis* eggs. After an initial asymptomatic incubation period of many years, humans may develop AE, which can be lethal if left untreated [6]. Diagnosis of probable AE is based on imaging techniques such as abdominal ultrasound, magnetic resonance, and computer tomography as well as detection of specific serum antibodies [7]. However, case confirmation requires histopathology or positive PCR on a fine-needle biopsy, which is particularly important in areas co-endemic for AE and cystic echinococcosis (CE). Since AE is a rare disease with a long incubation period of several years, identification and evaluation of potential risk factors (PRFs) associated with infection is difficult. Moreover, the duration of the asymptomatic or paucisymptomatic period and the associated nonspecific symptoms in humans contribute to the massive underreporting of AE. Although in some regions considerable attention is afforded to the disease by both the general public and the media, AE is not yet mandatorily notifiable in most affected countries [8].

Human infection depends on behavioural and socio-economic variables that favour close contact with *E*. *multilocularis* eggs and their inadvertent ingestion [9, 10]. Additionally, risk factors and the geographic distribution of AE may differ from country to country and more importantly between regions as the infection is influenced by both biotic and abiotic factors.

The objective of this study was to summarize evidence relating to both proven and potential risk factors associated with *E*. *multilocularis* human infection by conducting a systematic review (SR) and meta-analysis of studies in accordance with the Cochrane and PRISMA group guidelines. The results of this SR may help establish or improve existing surveillance systems to prevent human infection with *E*. *multilocularis*.

## Methods

### Search strategy, inclusion and exclusion criteria

This SR was essentially performed using the same methodology as in recently published SR on PRFs associated with human cystic echinococcosis [11]. Literature databases were searched using keywords associated with the Boolean operators AND/OR, the question mark (?) and the hash mark (#). The question mark (?) expanded the search by looking for words with similar prefixes using more than one letter (i.e. “echinococc?” was used to search for “echinococcus”, “echinococci”, “echinococcosis” and “echinococcoses”), whereas the hash mark (“#”) expanded the search by looking for words with similar prefixes using one letter (i.e. dog# was used to search for “dog” or “dogs”). The strategy developed in PubMed/Medline used queries for papers reporting abstracts on risk factors related to human AE. Thus, the final strings used for the search were [echinococcus multilocularis OR (echinococcus AND multilocularis) OR e# multilocularis OR alveolar echinococcosis OR a# echinococcosis] AND (risk factor# OR risk#) AND (human# OR people OR person OR man OR men OR women OR woman OR patient#).

The following inclusion criteria were used: primary research studies published from 1950 onwards or in press at the time of the search; articles published in English, German, French, Polish, Finnish, Dutch, Spanish or Italian; studies with a case-control, cross-sectional or cohort design. Articles were excluded if they contained only the results of descriptive studies (including case reports), if a data-driven assessment of PRFs for human infection with *E*. *multilocularis* was missing, if agents other than *E*. *multilocularis* (e.g. *Echinococcus granulosus* sensu lato) were investigated or if no difference was made between AE and CE. In addition, review articles, letters or editorials without original data, duplicated data and articles with full texts written in languages other than those mentioned above were not included.

### Study design: Search strategy and data extraction

This SR and meta-analyses followed the Cochrane and PRISMA (Preferred Reporting Items for SRs and Meta-analyses) Group guidelines [12]. The PRISMA 2009 checklist is provided as supporting information (S1 Checklist). The platform used for searching databases was STN International–Fiz Karlsruhe (http://www.fiz-karlsruhe.de/stn.html?&L=1). The first online electronic search was carried out on 5th November 2013 and updated on 11th February 2015 and again on 1st April 2016 to identify papers published since the initial search. The platform used to carry out the literature search included six bibliographic databases: Medical Literature Analysis and Retrieval System Online (MEDLINE), Excerpta Medica Database (EMBASE), Science Citation Index (SciSearch), Biological Abstracts (BIOSIS), Centre for Agricultural Bioscience International (CABI) and Google Scholar. Article selection was carried out using a three step process: first, duplicate articles were removed, then titles and abstracts were read taking the keywords into account, and finally full texts were screened for eligibility. Data were extracted from elegible studies using standardized Microsoft Excel tables (Microsoft Office 2010, version 14.0). The following data were recorded in the extraction tables: paper identification (ID, sub-ID, first author, year of publication, title, journal, volume, page numbers), brief study description, study design (case-control, cross-sectional and cohort study), year of study, geographic area, country and diagnostic method (ultrasound, surgery, molecular identification and serology), PRFs and quality assessment. Data were extracted by two independent researchers and any disagreements were resolved by consensus among the researchers using the standardized extraction forms to guarantee consistency and accuracy. Articles meeting the inclusion criteria were evaluated, and data relating to PRFs were extracted according to the following groups: association with dogs/cats, gender, age, familiar or ethnic clusters, living in a rural area, having a kitchen garden, occupation, food/water contact, hunting/ handling foxes and socio-cultural status. The terms for PRFs were used as referred to in the respective studies and were not merged, even if they appeared to be similar (e.g. dog ownership; allowed dog into the house; allowed dog into bedroom; was licked by dog; playing with dogs, brushed the dog´s fur; etc.). Data from studies based only on serology were extracted but due to the low sensitivity/specificity of this diagnostic approach, these studies were meta-analysed only when ultrasound results were also reported [13, 14].

All patient medical data analysed in this study were anonymised and extracted from publications, in which they were reported in an aggregated form as case or population counts. No individual patient data were used in this study. Approval from an ethical committee or institutional review board was not necessary for this research.

### Quality assessment

The quality assessment of the studies included in this SR was performed by two independent reviewers using the Newcastle-Ottawa Scale (NOS) according to the Cochrane Handbook for Systematic Reviews [15, 16]. If the assessments differed, they were discussed until a consensus had been reached. Studies were scored in three domains: (i) selection of study groups, (ii) their comparability and (iii) the ascertainment of either the exposure or outcome of interest for case-control or cohort studies, respectively. The "selection" category covers four properties: use of an adequate case definition, representativeness of the cases, selection of controls and the definition of controls. The "exposure" category consists of three properties: ascertainment of exposure, the use of the same method of ascertainment for cases and controls, and information on the non-response rate. A study could be awarded a maximum of one star for each item in the categories "selection" and "exposure". A maximum of two stars could be given for "comparability" (study controls for one factor/ for any additional factor), i.e. a maximum score of 4 (“selection”), 3 (“exposure”) and 2 (“comparability”) stars could be allocated leading to a maximum score of 9.

### Research evidence: Data- and meta-analysis

Statistical analysis was performed using the software Review Manager 5.2 (RevMan Version 5.2. Copenhagen: The Nordic Cochrane Centre, The Cochrane Collaboration, 2014; http://ims.cochrane.org/revman). The analyses were stratified in relation to the different PRFs reported in the included studies. Pooled odds ratios (OR) were used as a measure of effect and separately analysed for case-control and cross-sectional studies. Meta-analysis was performed when at least two studies reported data on the same PRF. OR, with the respective 95% confidence intervals (CI), were calculated for each potential risk or protective factor (if the factor was covered in more than two studies), and visualised using forest plots. The Cochran’s Q test was performed to assess the degree of heterogeneity between studies. *I*^2^ statistic was used to describe the percentage of total variation across studies. If the *p*-value of the Q test was < 0.05 and *I*^2^ was >50%, heterogeneity was inferred and a random-effect model was used. If heterogeneity was not detected, a fixed-effect model was adopted. Publication bias was quantified by inspection of funnel plots and computation of Egger [17] and Begg [18] test probability values. A meta-regression analysis was conducted for each potential risk or protective factor reported in more than two studies using SPSS for Windows (IBM Corp., 2013. IBM SPSS Statistics for Windows, Version 22.0, Armonk, Ney York, USA). Year of publication, total population and quality scores were considered as variables. For each analysis, a linear regression model was built by stepwise backward elimination of variables. The results of the analyses are presented with their beta coefficients and *p-*values. The threshold for statistical significance was set at *p*<0.05.

## Results

### Study selection process

**Fig 1:**
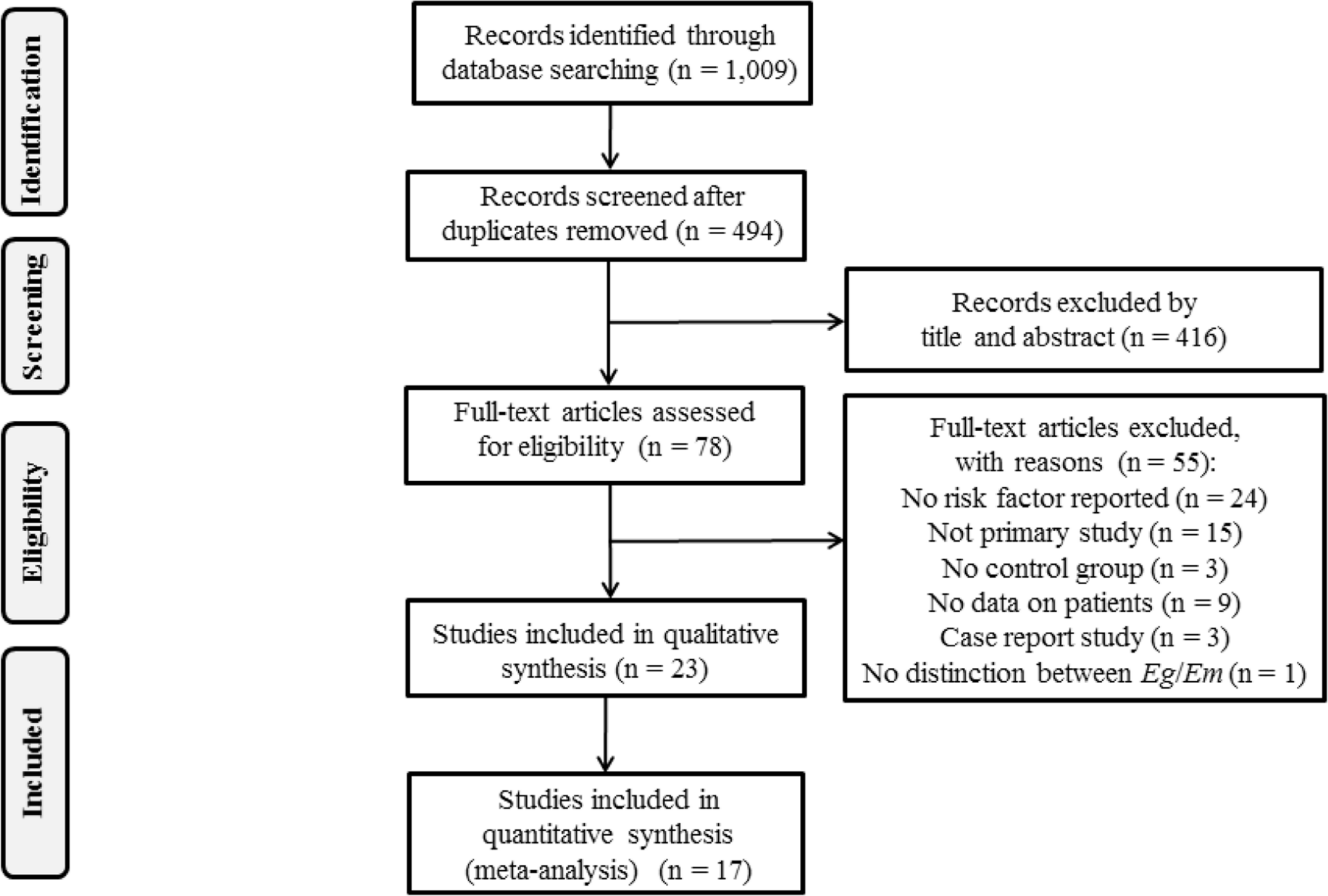
Literature search. The number of articles returned and examined at each stage of the research on potential risk factors associated with human alveolar echinococcosis (AE) is shown.

A total of 1,009 potentially relevant publications were identified. Following the removal of 515 duplicates generated between databases, the titles and abstracts of 494 papers were screened and a further 416 papers were excluded (Fig 1). The full texts of the remaining 78 publications were read, and 55 papers were excluded (S1 Table) based on the following: no risk factor was reported, the publication failed to be a primary study, no control group could be identified, no data on patients were reported, the publication was characterized as a case report or no distinction was made between *E*. *multilocularis* and *E*. *granulosus*. A total of 23 publications were found eligible for inclusion in this SR (S2 Table), of which 17 were subjected to meta-analysis to determine associations between human AE infection and potential risk or protective factors. The remaining 6 publications were unsuitable for meta-analysis as no OR for individual risk factors could be extracted. Of the 17 publications, data were separately extracted from case-control (n = 6) [19–24] and cross-sectional studies (n = 11) [10, 25–34] (S2 Table; S1 Flow Diagram). No cohort studies were identified. The geographical areas relevant to the 17 studies included in this review were China (n = 11) [10, 24–31, 33, 34], Central Europe (n = 5) [19–22; 32] and North America (n = 1) [23]. Papers used in the current SR were published between 1988 and 2013.

When studies (i.e. individual publications) were conducted on different groups of individuals (for example pastoral versus urban communities [10]) or in different areas (for example regions at risk versus regions not at risk of AE [21]), they were divided into sub-studies and each sub-study was analysed separately. Eventually, 8 and 13 sub-studies were identified for case-control and cross-sectional studies, respectively and each of these two groups were meta-analysed separately. The results of the meta-analyses (forest plots, OR and pooled OR with their respective 95% CI) and potential publication bias (funnel plots) were separately recorded for case-control (S1 Supplementary information) and cross-sectional studies (S2 Supplementary information). When the meta-analysis included only a small number of studies, it was not possible to assess publication bias using funnel plots.

The assessment of the quality of the studies included in this SR was performed using NOS through the implementation of a ‘star system’. Of the 6 case-control studies, 3 were allocated an 8-star rating [19, 22, 23] and the other 3 studies received a 9 [20], 4 [24] and a 2-star score [21]. Within the 11 cross-sectional studies, 5 received a 8-star rating [10, 26, 28–30], 3 a 6-star rating [25, 33, 34], 2 a 7-star rating [31, 32] and 1 a 9-star score [27].

### Potential risk factors: Meta-analysis of case-control studies

Sixteen PRFs were identified from case-control studies and meta-analyses were performed on six papers corresponding to eight sub-studies. Most studies originated from endemic areas in Central Europe, namely Austria (n = 1) [22], France (n = 1) [21], Germany (n = 1) [19], France-Germany-Switzerland (n = 1) [20]; North America (Alaska) (n = 1) [23] and Asia (People's Republic of China) (n = 1) [24]. All these retrospective studies were hospital-based and included control groups that were not affected by AE but had demographic characteristics similar to those of the AE patients.

The following groups of PRFs were investigated in this meta-analysis (PRFs are termed as reported in the publications): 4 pet-related (“dog ownership”, “allowed dog into the house”, “play with dogs”, “cat ownership”), 4 food-related (“ate mushrooms”, “consumption of wild vegetables and fruit”, “ate unwashed strawberries”, “having a kitchen garden”), 7 related to working or recreational activities in rural areas (“hunting”, “hunting / handling foxes”, “occupation: farmer”, “haymaking in meadows not adjacent to water”, “went to forests for vocational reasons”, “chewed grass”, “live in a rural area”) and one potentially protective genetic factor (“human leukocyte antigen, HLA”).

Eight PRFs were associated with statistically significant increased OR (test for overall effect, *p*<0.05): “dog ownership” (OR 2.50; 95% CI 1.73–3.62; *p*<0.00001), “cat ownership” (OR 2.63; 95% CI 1.42–4.85; *p*<0.002), “having a kitchen garden” (OR 5.21; 95% CI 2.65–10.22; *p*<0.00001), “occupation: farmer” (OR 4.50; 95% CI 2.74–7.39; *p*<0.00001), “haymaking in meadows not adjacent to water” (OR 3.50; 95% CI 1.63–7.55; *p* = 0.001), “went to forests for vocational reasons” (OR 2.61; 95% CI 1.13–6.05; *p*<0.03), “chewed grass” (OR 3.20; 95% CI 1.65–6.20; *p* = 0.00006) and “handling foxes” (OR 2.27; 95% CI 1.35–3.81; *p* = 0.002).The PRF “HLA” was statistically significant (*p* = 0.003) with an OR of 0.50 (95% CI: 0.32–0.80), which indicated that particular human leucocyte antigens may be protective against AE infection.

For seven PRFs, the results were not statistically significant: “allowed dog into the house” (OR 1.80; 95% CI 0.90–3.62; *p* = 0.10), “play with dogs” (OR 1.24; 95% CI 0.39–3.91; *p* = 0.71), “living in a rural area” (OR 3.07; 95% CI 0.83–11.37; *p* = 0.09), “ate unwashed strawberries” (OR 1.39; 95% CI 0.87–2.23; *p* = 0.17), “hunting” (OR 1.13; 95% CI 0.69–1.83; *p* = 0.63), “ate wild vegetables and fruit” (OR 1.38; 95% CI 0.90–2.10; *p* = 0.14) and “ate mushrooms” (OR 0.72; 95% CI 0.38–1.39; *p* = 0.33). PRFs meta-analyzed for case-control studies are summarized in Table 1. Forest plots, funnel plots and single weight of each publication contributing to the overall risk factors are presented in S1 supplementary information.

**Table 1:** Meta-analysis of potential and protective risk factors identified in case-control studies on human alveolar echinococcosis.

POTENTIAL RISK FACTOR	Effect model	Odds ratio (95% CI)	Overall effect	Number of sub-studies	Number of participants in studies	References
**Dog ownership**	**M-H, Fixed**	**2.50 [1.73–3.62]**	**p<0.00001**	**5**	**1,068**	**19, 21, 22, 23**
* Allowed dog into the house	M-H, Fixed	1.80 [0.90–3.62]	p = 0.10	2	216	19, 23
*Play with dogs	M-H, Random	1.24 [0.39–3.91]	p = 0.71	2	216	19, 23
**Cat ownership**	**M-H, Fixed**	**2.63 [1.42–4.85]**	**p = 0.002**	**2**	**265**	**19, 22**
* Living in a rural area	M-H, Random	3.07 [0.83–11.37]	p = 0.09	3	803	21, 23
**Having a kitchen garden**	**M-H, Fixed**	**5.21 [2.65–10.22]**	**p<0.00001**	**2**	**746**	**21**
**Occupation: farmer**	**M-H, Fixed**	**4.50 [2.74–7.39]**	**p<0.00001**	**4**	**1,011**	**19, 21, 22**
**Haymaking in meadows not adjacent to water**	**M-H, Fixed**	**3.50 [1.63–7.55]**	**p = 0.001**	**2**	**238**	**19, 22**
**Went to forests for vocational reasons**	**M-H, Fixed**	**2.61 [1.13–6.05]**	**p = 0.03**	**2**	**266**	**19, 22**
*Ate unwashed strawberries	M-H, Fixed	1.39 [0.87–2.23]	p = 0.17	4	1,006	19, 21, 22
**Chewed grass**	**M-H, Fixed**	**3.20 [1.65–6.20]**	**p = 0.00006**	**2**	**252**	**19, 22**
* Hunting	M-H, Fixed	1.13 [0.69–1.83]	p = 0.63	5	1,064	19, 21, 22, 23
**Handling foxes**	**M-H, Fixed**	**2.27 [1.35–3.81]**	**p = 0.002**	**4**	**959**	**19, 21, 23**
§ Ate mushrooms	M-H, Fixed	0.72 [0.38–1.39]	p = 0.33	2	255	19, 22
* Ate wild vegetables and fruit	M-H, Fixed	1.38 [0.90–2.10]	p = 0.14	5	1,046	19, 21, 22, 23
**HLA (protective factor)**	**M-H, Fixed**	**0.50 [0.32–0.80]**	**p = 0.003**	**2**	**743**	**20, 24**

**Bold**: significantly increase of odds ratio.

*: increased odds ratio, but not statistically significant

§: influence on infection risk uncertain.

### Meta-analysis of cross-sectional studies

Meta-analyses performed on eleven cross-sectional studies corresponding to thirteen sub-studies revealed thirteen PRFs. Most studies originated from endemic areas in the People's Republic of China, namely, Ningxia Hui autonomous region (n = 2) [29, 34], Sichuan province (n = 4) [10, 25, 26, 33], Gansu province (n = 2) [30, 31], Qinghai province (n = 1) [28], Sichuan and Qinghai provinces (n = 1) [27] and one from Europe (Germany) [32]. All studies were community-based ultrasonography surveys.

PFRs were grouped as follows: two PFRs were dog-related (“dog ownership”, “play with dogs”), 2 were linked to drinking water (“source of drinking water other than well or tap”, “drinking non-boiled water”), 3 were related to working activities (“occupation: farmer”, “occupation: herder”, “hunting/ handling foxes”), 4 were socio-culturally related (“ethnic group: Tibetan”, “low income”, “low education”, “hand wash before eating”) and two could not be attributed to a specific group (“gender: female”, “age over 20 years”).

“Low education” was differentiated into having no education or attended primary school versus secondary school, college or higher education. “Low income” was defined as having a salary of less than 5.000 Chinese Yuans (RMB) per year (exchange rate 2005: 8.1 Yuan = 1 US $).

Nine PRFs were statistically significant and exposure was associated with increased OR (test for overall effect, *p*<0.05): “dog ownership” (OR 1.95; 95% CI 1.52–2.51; *p*<0.00001), “play with dogs” (OR 3.48; 95% CI 2.20–5.52; *p*<0.00001), “gender: female” (OR 1.66; 95% CI 1.31–2.10; *p*<0.00001), “age over 20 years” (OR 3.65; 95% CI 1.15–11.62; *p*<0.03), “ethnic group: Tibetan” (OR 2.03; 95% CI 1.56–2.63; *p*<0.00001), “low income” (OR 3.92; 95% CI 2.42–6.36; *p*<0.00001), “source of drinking water other than well or tap” (OR 1.81; 95% CI 1.52–2.17; *p*<0.001), “occupation: herder” (OR 2.20; 95% CI 1.51–3.19; *p*<0.00001) and “low education” (OR 4.81; 95% CI 2.73–8.48; *p*<0.00001).

The PRF “drinking non-boiled water” was protective (OR<1) against infection at a statistically significant level (OR 0.63; 95% CI 0.48–0.84; *p* = 0.002). For three PRFs, it was not possible to determine their effect (protective, risk or none): “hand washing before eating” (OR 4.90; 95% CI 0.80–29.87; *p* = 0.08), “occupation: farmer” (OR 1.16; 95% CI 0.31–4.35; *p* = 0.82) and “hunting / handling foxes” (OR 1.25; 95% CI 0.71–2.20; *p* = 0.44).

The PRFs meta-analyzed for cross-sectional studies are summarized in Table 2. Forest plots, funnel plots and single weight of each publication contributing to the overall risk factors are presented in S2 supplementary information.

**Table 2:** Meta-analysis of potential and protective risk factors identified in cross-sectional studies on human alveolar echinococcosis.

POTENTIAL RISK FACTOR	Effect model	Odds ratio (95% CI)	Overall effect	Number of sub-studies	Number of participants	References
**Dog ownership**	**M-H, Fixed**	**1.95 [1.52–2.51]**	**p<0.00001**	**4**	**12,571**	**26, 29, 33, 34**
**Play with dogs**	**M-H, Fixed**	**3.48 [2.20–5.52]**	**p<0.00001**	**3**	**5,916**	**10, 33**
* Hand wash before eating	M-H, Random	4.90 [0.80–29.87]	p = 0.08	3	5,348	10, 33
**Gender: female**	**M-H, Random**	**1.66 [1.31–2.10]**	**p<0.00001**	**10**	**42,812**	**10, 25–27, 29–34**
**Age over 20 years**	**M-H, Random**	**3.65 [1.15–11.62]**	**p = 0.03**	**8**	**24,988**	**10, 25, 26, 28, 31–33**
**Ethnic group: Tibetan**	**M-H, Fixed**	**2.03 [1.56–2.63]**	**p<0.00001**	**4**	**25,952**	**10, 27, 29, 34**
**Low income**	**M-H, Fixed**	**3.92 [2.42–6.36]**	**p<0.00001**	**2**	**4,124**	**10**
**Source of drinking water other than well or tap**	**M-H, Fixed**	**1.81 [1.52–2.17]**	**p = 0.001**	**5**	**23,714**	**10, 29, 33, 34**
* Occupation: farmer	M-H, Random	1.16 [0.31–4.35]	p = 0.82	5	17,878	10, 26, 27, 29, 34
**Occupation: herder**	**M-H, Random**	**2.20 [1.51–3.19]**	**p<0.00001**	**5**	**21,045**	**10, 26, 27, 29**
**Drink un-boiled water**	**M-H, Fixed**	**0.63 [0.48–0.84]**	**p = 0.002**	**2**	**7,096**	**10, 29**
* Hunting / handling foxes	M-H, Random	1.25 [0.71–2.20]	p = 0.44	3	9,442	26, 29, 33
**Low education**	**M-H, Fixed**	**4.81 [2.73–8.48]**	**p<0.00001**	**2**	**5,297**	**10**

**Bold**: significantly increase of odds ratio.

* Increased odds ratio, but not statistically significant

## Discussion

This SR and meta-analysis summarized the current evidence on PRFs associated with human AE infection. The initial search revealed more than 1,000 studies published during the last 65 years, a fact that emphasizes the broad relevance of this topic. The studies included in this SR refer to a total of 3,091 AE cases. The number of AE cases per study varies from one [32] to 577 [27]. The largest control group comprised 15,614 persons [27]. We cannot rule out, however, that some persons were enrolled in more than one study as all data were used in an anonymised form. This might have been the case in the study of Eiermann et al. (1998), which included 64 patients from France and 34 patients from Germany [20]. The same patients may have also been enrolled in the studies of Piarroux (2013) [21] with 180 AE cases from France and Kern (2004) with 40 AE cases from Germany [19]. However, even if the same patients were included, the aim, approach and methods of the studies were different. While the study of Eiermann et al. (1998) [20] focused on immunological markers, the two other studies [19, 21] analysed environmental risk factors. So, inclusion of identical people in different studies investigating different PRFs does not bias the results of our meta-analysis.

During the SR process, papers were evaluated by quality assessment tools, but similar to previous SR, e.g. [35], it was not possible to evaluate the validity of diagnostic techniques or to differentiate between “possible”, “probable” or “confirmed” diagnosis.

In general, dog-related PRFs for human AE are common worldwide. Indeed, the results seen here indicate that dog ownership is the most clearly established risk factor for acquiring human AE. It seems possible that a domestic cycle of *E*. *multilocularis* exists at least in some regions, which may facilitate transmission of the parasite to humans and therefore increase the risk of human AE. The presence of *E*. *multilocularis*-infected dogs seems to play a central role for the occurrence of human AE in China [36], while foxes, if present in affected regions, can act as additional definitive host for the parasite. In Europe, the role of dogs is less clear. Although it is undisputable that dogs may play a role in AE transmission in Europe, dog-related PRFs could represent confounders in areas where transmission might occur mainly through infected foxes. In these predominantly rural areas, people may be more frequently exposed to dog-related PRF, but the infection pressure might in fact be increased due to the presence of a large biomass of infectious *E*. *multilocularis* eggs excreted by foxes into the environment. However, we cannot exclude potential pathways of transmission related to the behavior of dogs, even when they are not infected. For instance, dogs commonly engage in a behavior known as scent-rolling: On finding any source of strong or unique-smelling, such as urine or faeces (for instance dispersed by positive foxes) or any other pungent odor that is not a regular scent within their territory, they roll their face and body in it, thus transferring the odor to their coat. Such behavior can increase the probability of eggs sticking onto their fur. We hypothesize that non-infected dogs roaming freely in endemic areas can act as carriers of *E*. *multilocularis* eggs, thus increasing the potential risk of human infection.

It should also be noted that “being a dog owner” probably comprehensively includes variable human/dog behaviours reflecting a number of different PRFs. For instance, Kern and colleagues [19] identified an increase in odds of AE infection depending on factors such as “leaving the dog in the garden unattended”, “dog killing the game”, “allowed dog into the house“, “walked dog without leash“, “dog ate mice”and “had dog dewormed infrequently or never“. The use of tailored questionnaires in endemic areas focusing on specific PRFs related to dogs in order to elucidate pathways of transmission mediated by human/dog behaviour, are strongly encouraged.

Cats have occasionally been found infected with *E*. *multilocularis*, and a zoonotic potential cannot be totally ruled out, however they are considered as less suitable definitive hosts [37]. In a recently published study, a high worm burden was detected in cats, but it comprised only of immature worms, which indicated that the fecundity of *E*. *multilocularis* in cats was rather low [38]. In fact, *E*. *multilocularis* eggs derived from adult tapeworms harboured by cats failed to develop into metacestodes when used to experimentally infect mice [1]. More recently, faeces, identified to be of feline origin using genetic analysis, was found to contain eggs of *E*. *multilocularis* [39]. Additionally, cats may behave in a way that can increase the probability of transmitting the infection to humans, e.g. by *E*. *multilocularis* eggs sticking onto their fur. Interestingly, a significant increase in the risk of human AE have been identified for the cat-associated PRFs “left unattended outdoors” and “ate mice” [19].

Food-related risk factors such as eating mushrooms, wild berries or unwashed strawberries illustrate that vegetables and fruit, growing close to the ground may become contaminated with *E*. *multilocularis* eggs shed by infected definitive hosts, which may lead to human exposure to *E*. *multilocularis* if eaten raw or undercooked. The same may hold true for water, especially if contaminated surface water is used for drinking. The relative importance of food- or waterborne AE infection among other risk factors will depend, however, on general sanitation practices, e.g. washing wild berries or adequately treating natural surface water. The debate regarding the importance of unwashed contaminated fresh fruit, vegetables and mushrooms in the transmission of *E*. *multilocularis* is still ongoing [40–42]. Our meta-analysis illustrates that the chance of AE transmission through the ingestion of food and water contaminated with *E*. *multilocularis* eggs does indeed exist, but it is important to note that food- and water-borne PRFs do not significantly increase the risk of infection. Similar findings have been recently reported for CE [11], although it should be emphasized that water- and food-borne transmission seems to be more evident for AE as compared to CE. This finding is also supported by a recent SR on foodborne parasitic diseases, in which the percentage of foodborne CE and AE (reported as foodborne_DALYs/total_DALYs x100) was estimated to be 21% and 48%, respectively [43].

PRFs related to working or recreational activities in endemic areas may also point to an increased exposure of people to *E*. *multilocularis* eggs present on the fur of infected definitive hosts or in the environment, in particular in gardens and in areas used for agricultural purposes. In fact, not only “being a farmer” or “went to forests for vocational reasons” seems to be a significant PRF but also activities increasing the probability of ingesting eggs inadvertently such as “chewed grass” and “hunting / handling foxes”.

The fact that particular HLA types appeared to be a potentially protective factor against AE infection may indicate that the susceptibility of humans to infection with *E*. *multilocularis* has a genetic component and that humans with these HLA antigens may be less likely to contract the disease or to develop clinically apparent AE [44], but this aspect may have to be studied in more detail. Some socio-cultural factors (for example, poverty as illustrated by low income and low level of education or ethnic background such as the increased risk of Tibetans of contracting AE) may also play an important role in AE transmission.

The literature research carried out in this study identified several publications, which contained information on PRFs presented in formats that precluded their extraction or the computation of OR. These PRFs included human immunosuppression [45], landscape composition [46, 47], landscape and climatic characteristics [48], age, occupation (housewives), dog-ownership, consumption of wild vegetables and fruit [49], farming [50–52], presence of stray dogs and stray cats [51], hunting [53], changes in the ecology of wild hosts [54] and family clusters [55]. In addition, approaches used in meta-analysis may lead to the detection of statistically significant PRFs, which may nevertheless be spurious. This is why we explicitly refer to “potential” risk factors. Despite existing limitations of the SR approach, e.g. potential bias due to exclusion of studies that contain information in a format that is not compatible with SR or meta-analysis and the “pooling” of data from different studies, which may not always be sufficiently comparable, SR and meta-analysis are generally accepted as a tool in evidence-based science.

In this literature review, age, gender and dog ownership have been identified as PRFs, however it should be emphasised that some of these may represent confounders. A critical discussion on confounding, intrinsic limits and advantages of case-control and cross-sectional studies on CE, that can be applied to AE, has been recently published [11].

It is also noteworthy that including data originating from different geographical regions (for example from China and Europe) in a meta-analysis, harbours risks and uncertainties, which must be taken into account when interpreting the results. Indeed, factors for acquiring AE in Europe might differ from those in central Asia, which may be due for example to different living conditions (hygienic conditions in rural areas, income etc.) and habits (most European citizens are more or less sedentary and keep dogs or cats as pets, which may reduce the risk to prey on infected intermediate hosts). The majority of case-control (4/6) and cross-sectional (11/12) studies included in this SR originated from Europe and China, respectively. It was therefore reasonable to suspect that some of the PRFs that were significantly associated with an increase in the risk of developing AE may reflect certain sociocultural determinants. It should also be stressed that results obtained in the meta-analysis could be biased by the fact that a large proportion (91%) of the global burden of AE originates from China [9]. In order to clarify this, we performed a meta-analysis, in which we compared case-control sub-studies from Europe (n = 6) with those from all regions (Europe, n = 6; Alaska, n = 1; China, n = 1) and cross-sectional sub-studies from China (n = 12) with those reported globally (China, n = 12; Europe, n = 1). Since the results of these analyses suggested that relevant PRFs remained the same both in Europe and China, we decided to aggregate the data for these geographical areas, thus increasing the power of the statistical analysis. The absence of a difference between the PRFs observed here may, however, be related to the disproportion in the number of individuals enrolled in the studies originating from these areas.

Our SR is limited to articles published in English, German, French, Polish, Finnish, Dutch, Spanish or Italian. Papers published exclusively in Chinese, Russian and Turkish (i.e. without an abstract in any of the languages listed in the manuscript) were not taken into account, since these languages were not understood in the consortium. However, relevant peer-reviewed studies are normally published in English or include an English abstract. Although Chinese was excluded from the language search, the highest number of retrieved studies used for data extraction and meta-analysis were from China (n = 9), but they were published in English. Furthermore, a study on the effect of language restriction on SR-based meta-analyses in conventional medicine found no evidence of bias as a result of language restriction [56].

This SR provides an up-to-date account of PRFs for human AE based on the meta-analyses of the available literature since 1950. This study may constitute the basis for designing and improving programmes aimed at the control and prevention of this severe human disease.

## Supporting information

S1 Checklist PRISMA checklist reporting items for this systematic reviews and meta-analyses. (DOC)

S1 Table List of studies excluded from the systematic review after full text screening. (DOCX)

S2 Table List of studies included in the systematic review after full text screening. (DOCX)

S1 Flow Diagram PRISMA flow diagram. (DOC)

S1 Supplementary information Case-control studies reporting forest and funnel plot analysis on single potential risk factors. (DOCX)

S2 Supplementary information Cross-sectional studies reporting forest and funnel plot analysis on single potential risk factors. (DOCX)
