# Supplementary material for: Potential risk factors associated with human alveolar echinococcosis: Systematic review and meta-analysis
Source: PLoS Negl Trop Dis. 2017 Jul 17;11(7):e0005801. doi: 10.1371/journal.pntd.0005801 (PMC5531747; doi:10.1371/journal.pntd.0005801)
Supplement: S1 Table — (DOCX) [file pntd.0005801.s002.docx]

# Supplementary Table 1. List of studies excluded from the systematic review after full text screening.

| First Author's Name | Reference | Article Title | Reason for excluding |
| --- | --- | --- | --- |
| D. Antolova et al. | Eurosurveillance. 2014 19(34):arn. 20882. | Alveolar echinococcosis in a highly endemic area of northern Slovakia between 2000 and 2013. | No risk factors described |
| M. Aubert et al. | Recueil de Medecine Veterinaire de l’Ecole d’Alfort. 1987 163(10):839–843. | Parasitism of red fox *Vulpes vulpes* by *Echinococcus multilocularis* in Lorraine France and their consequences on human contamination. | No risk factors described |
| P. Burlet et al. | Parasites & vectors. 2011 4:6. | Age season and spatio-temporal factors affecting the prevalence of *Echinococcus multilocularis* and *Taenia taeniaeformis* in *Arvicola terrestris.* | No data on human patients |
| A. Chauchet et al. | Journal of Hepatology. 2013 58(Suppl.1):S381. | Emergence of a new opportunistic infection in europe: Hepatic alveolar echinococcosis . A fifty-case report. | No risk factors described |
| P.S. Craig et al. | Parasitology international. 2006 55(Suppl):S221–5. | Epidemiology of human alveolar echinococcosis in China. | No primary research |
| M.P. Czarkowski and E. Golab | Przeglad epidemiologiczny. 2013 67(2):263–6 365–7. | Invasive tapeworm infections in Poland in 2011. | No risk factors described |
| F.M. Danson, et al. | Proceedings of the NATO Advanced Research Workshop on cestode zoonoses: echinococcosis and cysticercosis: an emergent and global problem Poznan Poland 2002:237–248. | Satellite remote sensing and geographical information systems for risk modelling of alveolar echinococcosis. | No primary data on PRF reported |
| F.M. Danson et al. | Photogrammetric Engineering and Remote Sensing. 2004 70(3):359–366. | Landscape dynamics and risk modelling of human alveolar echinococcosis. | Model no primary data on PRF reported |
| F.M. Danson et al. | Parasitology international. 2006 55(Suppl):S227–31. | Spatial modelling and ecology of *Echinococcus multilocularis* transmission in China. | Model no primary data on PRF reported |
| G. Duscher et al. | Wiener Tierarztliche Monatsschrift. 2005 92(1):16–20. | *Echinococcus multilocularis* in foxes in Vienna and surrounding territories. | No risk factors described |
| J. Eckert et al. | Clinical microbiology reviews. 2004 17(1):107–35. | Biological epidemiological and clinical aspects of echinococcosis a zoonosis of increasing concern. | No primary research |
| J. Gawor et al. | Przeglad epidemiologiczny. 2004 58(3):459–65. | Risk of alveococcosis for humans in Poland. | No risk factors described |
| J. Gawor | Przeglad epidemiologiczny. 2011 65(3):465–70. | Potential risk factors for alveolar echinococcosis in humans in Poland. | No risk factors described |
| P. Giraudoux et al. | Parasitology International. 2006 55[S]:S237-S246. | Transmission ecology of *Echinococcus multilocularis*: What are the ranges of parasite stability among various host communities in China? | No risk factors for human infection described |
| E. GolAb and M.P. Czarkowski | Przeglad epidemiologiczny. 2014 68(2):279–82 379–81. | Echinococcosis and cysticercosis in Poland in 2012. | No distinction between AE and CE |
| B. Gottstein et al. | Applied parasitology. 1996 37(2):129–36. | Investigations on a Swiss area highly endemic for *Echinococcus*. | No specific risk factors for human infection described |
| B. Gottstein et al. | Schweizerische Medizinische Wochenschrift. 1997 127(39):1629. | Risk assessment of infection with *Echinococcus multilocularis* in a highly endemic focus of Switzerland. | No specific risk factors for human infection described |
| B. Gottstein et al. | Emerging infectious diseases. 2001 7(3):408–12. | Is high prevalence of *Echinococcus multilocularis* in wild and domestic animals associated with disease incidence in humans? | No specific risk factors for human infection described |
| A.J. Graham et al. | Acta Tropica. 2004 91(3):267–278. | Ecological epidemiology: landscape metrics and human alveolar echinococossis. | No specific risk factors for human infection described |
| J. Han et al. | PLoS ONE. 2015 10(7):1-11. | A newly discovered epidemic area of *Echinococcus multilocularis* in West Gansu province in China | No data on human patients |
| U. Immelt et al. | Tieraerztliche Umschau. 2009 64(4):199–212 109. | Investigation of *Echinococcus multilocularis* in red foxes and their possible relationship to human alveolar echinococcosis. | Case report |
| H. Ishikawa et al. | Parasitology research. 2003 91(6):444–51. | A model for the transmission of *Echinococcus multilocularis* in Hokkaido Japan. | No risk factors described |
| A. Ito et al. | Acta Tropica. 2003 86(1):3–17. | Control of echinococcosis and cysticercosis: a public health challenge to international cooperation in China. | No risk factors described |
| E.J. Jenkins et al. | Advances in Parasitology. 2013 82:33–204. | Tradition and Transition: Parasitic Zoonoses of People and Animals in Alaska Northern Canada and Greenland. | No risk factors described |
| P. Kern et al. | American Journal of Tropical Medicine and Hygiene. 2003 69(3):366–367. | Risk factors for human alveolar Echinococcosis in Germany. | Original data reported in Kern et al. 2004 (included) |
| P. Kern et al. | Emerging Infectious Diseases. 2003 9(3):343–349. | European echinococcosis registry: Human alveolar echinococcosis Europe 1982–2000. | Does not contain controls |
| A. Koenig and T. Romig | Wildlife Biology. 2010 16(3):258–266. | Fox tapeworm *Echinococcus multilocularis* an underestimated threat: a model for estimating risk of contact. | No risk factors described |
| W. Kratzer and P. Kern | Internistische Praxis. 2001 41(3):693. | Risk of transmission of fox tapeworm. | No primary research |
| I. Lewis Fraser et al. | PLoS Neglected Tropical Diseases. 2014 8(3):e2731. | Dynamics of the force of infection: insights from *Echinococcus multilocularis* infection in foxes. | No risk factors described |
| J. Ma et al. | Veterinary parasitology. 2015 207(1–2):44–8. | Surveillance of *Echinococcus* isolates from Qinghai China. | No data on human patients |
| C.G. Marston et al. | Applied Geography. 2014 55:176–183. | A random forest approach for predicting the presence of *Echinococcus multilocularis* intermediate host *Ochotona* spp. presence in relation to landscape characteristics in western China. | No risk factors described |
| A. Muehling and P. Kimmig | Zentralblatt fuer Bakteriologie Mikrobiologie und Hygiene Abt 1 Originale B Hygiene Umwelthygiene Krankenhaushygiene Arbeitshygiene Praeventive Medizin. 1985 181(1–2):24. | Increased risk of infection by *Echinococcus multilocularis* for people living in the endemic region Schwaebische-Alb West Germany? | No primary research |
| W.L. Nahorski et al. | PloS Neglected Tropical Diseases. 2013 7(1):1-8 e1986. | Human Alveolar Echinococcosis in Poland: 1990-2011. | No control group |
| H.D. Nothdurft et al. | Deutsche medizinische Wochenschrift. 1995 120(34–35):1151–5. | Epidemiology of echinococcosis in Bavaria. | No control group |
| W. Poeppl et al. | Vector borne and zoonotic diseases (Larchmont N.Y.). 2013. | Exposure to *Echinococcus multilocularis* *Toxocara canis* and *Toxocara cati* in Austria: A Nationwide Cross-Sectional Seroprevalence Study. | No risk factors described |
| R.L. Rausch and F.H. Fay | Proceedings of the NATO Advanced Research Workshop on cestode zoonoses: echinococcosis and cysticercosis: an emergent and global problem Poznan Poland. 2002:309–325. | Epidemiology of alveolar echinococcosis with reference to St Lawrence Island Bering Sea. | No primary research |
| E.C. Renner-Schneiter et al. | Hepatology. 2000 32(4 Pt.2):625. | Does the risk of *Echinococcus multilocularis* (EM) infection increase with increasing populations of infected foxes in Swiss cities? A retrospective analysis of the cohort of the Swiss Echinococcosis Study Group (SESG) 1976–1999. | No risk factors described |
| J.M. Rijks et al. | Journal of comparative pathology. 2016 Vol.155:S75-S97. | Disease risk assessments involving companion animals: an overview for 15 selected pathogens taking a European perspective. | No primary research |
| T. Romig et al. | Deutsche Tierarztliche Wochenschrift. 1999 106(8):352-357. | Current data on the geographical distribution and epidemiology of *Echinococcus multilocularis*. | No primary research |
| Z. Said-Ali et al. | Parasitology. 2013 140(13):1693–700. | Detecting nested clusters of human alveolar echinococcosis. | No risk factors described |
| J.M. Schurer et al. | PLoS Neglected Tropical Diseases. 2015 9(7): 1-15. | Echinococcosis: an economic evaluation of a veterinary public health intervention in rural Canada. | Case report |
| S.B. Siko et al. | Parasitology research. 2011 108(5):1093–7. | *Echinococcus multilocularis* in south-eastern Europe (Romania). | No risk factors described |
| A.K. Uzunlar et al. | East African medical journal. 2003 80(8):395-7. | Echinococcosis multilocularis in south-eastern Anatolia Turkey. | No control group |
| J.F. Viel et al. | The American journal of tropical medicine and hygiene. 1999 61(4):559–65. | Water vole (*Arvicola terrestris* scherman) density as risk factor for human alveolar echinococcosis. | Case report |
| Q. Wang et al. | Emerging Infectious Diseases. 2006 12(6):1008–1010. | Pasture types and *Echinococcus multilocularis* Tibetan Communities. | No data on human patients |
| Q. Wang et al. | Chinese Medical Journal. 2007 120(3):237–242. | Impact of overgrazing on the transmission of *Echinococcus multilocularis* in Tibetan pastoral communities of Sichuan Province China. | No data on patients |
| Q. Wang et al. | Chinese Medical Journal. 2010 123(1):61–67. | Grass height and transmission ecology of *Echinococcus multilocularis* in Tibetan communities China. | No data on patients |
| Q. Wang et al. | Infectious diseases of poverty. 2014 3(1):3. | Review of risk factors for human echinococcosis prevalence on the Qinghai-Tibet Plateau China: a prospective for control options. | No primary research |
| N. Yamamoto et al. | Annals of tropical medicine and parasitology. 2001. 95(7):689-96. | Risk factors for human alveolar echinococcosis: a case-control study in Hokkaido Japan. | No data on patients |
| Y.R. Yang et al. | Journal of Tropical Medicine and Hygiene. 2006 75(5) Suppl. S:205. | Cystic and alveolar echinococcosis transmission and risk factors in Ningxia Hui Autonomous Region of China: Current situation and evolution. | No primary research |
| Y.R. Yang, et al. | Tropical medicine & international health. 2008 13(8):1086–94. | Serological prevalence of echinococcosis and risk factors for infection among children in rural communities of southern Ningxia China. | No data on human patients |
| Y.R. Yang et al. | Parasites and Vectors. 2012 5(146). | Impact of anthropogenic and natural environmental changes on *Echinococcus* transmission in Ningxia Hui Autonomous Region the People‘s Republic of China. | No primary research |
| R.Y. Yu et al. | American Journal of Tropical Medicine and Hygiene. 2006 74(3):487–494. | Unique family clustering of human echinococcosis cases in a Chinese community. | No data on human patients |
| W. Zhang et al. | Acta tropica. 2015 141(Pt B):235–43. | Epidemiology and control of echinococcosis in central Asia with particular reference to the People‘s Republic of China. | No primary research |
| H.X. Zhou et al. | Annals of tropical medicine and parasitology. 2000 94(7):715-29. | Epidemiology of alveolar echinococcosis in Xinjiang Uygur autonomous region China: a preliminary analysis. | No risk factor described |
