# Supplementary material for: Potential risk factors associated with human alveolar echinococcosis: Systematic review and meta-analysis
Source: PLoS Negl Trop Dis. 2017 Jul 17;11(7):e0005801. doi: 10.1371/journal.pntd.0005801 (PMC5531747; doi:10.1371/journal.pntd.0005801)
Supplement: S2 Table — (DOCX) [file pntd.0005801.s003.docx]

# Supplementary Table 2. List of studies included in the systematic review after full text screening.

| TITLE | REFERENCE | FIRST AUTHOR'S NAME | YEAR |
| --- | --- | --- | --- |
| Increased incidence and characteristics of alveolar echinococcosis in patients with immunosuppression-associated conditions. | Clinical infectious diseases: an official publication of the infectious diseases society of America. 59(8):1095–104. | A. Chauchet et al. | 2014 |
| A large focus of alveolar echinococcosis in central China. | **Lancet. 340(8823):826–31.** | **P.S. Craig et al.** | **1992** |
| An epidemiological and ecological study of human alveolar echinococcosis transmission in south Gansu, China. | **Acta tropica. 77(2):167–77.** | **P.S. Craig et al.** | **2000** |
| Zoonoses, seroepidemiological examination of different persons for selected contact zoonoses: seroprevalences, risk factors and preventative measures. | Fleischwirtschaft. 82(1):101–104. | A. Deutz et al. | 2002 |
| HLA and alveolar echinococcosis. | **Tissue antigens. 52(2): 124–9.** | **T.H. Eiermann et al.** | **1998** |
| Drivers of *Echinococcus multilocularis* transmission in China: small mammal diversity, landscape or climate? | **PLoS neglected tropical diseases. 7(3):e2045.** | **P. Giraudoux et al.** | **2013** |
| Vulpine tapeworm (*Echinococcus multilocularis*) infection (alveolar echinococcosis) in farmers as an occupational disease in accordance with BeKV no. 3102.  Risk factors for alveolar echinococcosis in humans. | Arbeitsmedizin Sozialmedizin Umweltmedizin 30(5):203–206.  **Emerging infection disease. 10(12):2088–93.** | S. Harbarth et al.  **P. Kern et al.** | 1995  **2004** |
| Increased risk of infection by *Echinococcus multilocularis* for people in the endemic “Schwaebische Alb” region? | Zentralblatt fur Bakteriologie, Mikrobiologie und Hygiene. 1. Abt. Originale B, Hygiene. 181(1–2):184–96. | P. Kimmig and A. Muhling | 1985 |
| Domestic pets as risk factors for alveolar hydatid disease in Austria. | **American journal of epidemiology. 147(10):978–81.** | **P. Kreidl et al.** | **1998** |
| HLA-DRB1 allele in 35 patients with alveolar echinococcosis in Gansu Province of China. | **Chinese medical journal. 116(10):1557–60.** | **F. Li et al.** | **2003** |
| Landscape and climatic characteristics associated with human alveolar echinococcosis in France, 1982 to 2007. | Euro Surveillance. Vol. 20(18). | M. Piarroux et al. | 2015 |
| Populations at risk for alveolar echinococcosis, France. | **Emerging infectious diseases. 19(5):721–8.** | **M. Piarroux et al.** | **2013** |
| Landscape composition and spatial prediction of alveolar echinococcosis in southern Ningxia, China. | **PLoS neglected tropical diseases. 2(9):arn. e287.** | **D.R.J. Pleydell et al.** | **2008** |
| An epidemiologic survey of human alveolar echinococcosis in southwestern Germany. Romerstein Study Group. | **The American journal of tropical medicine and hygiene. 61(4):566–73.** | **T. Romig et al.** | **1999** |
| Echinococcosis on the Tibetan Plateau: prevalence and risk factors for cystic and alveolar echinococcosis in Tibetan populations in Qinghai Province, China. | **Parasitology. 127:S109–20.** | **P.M. Schantz et al.** | **2003** |
| Seroepidemyology of human Alveolar Echinococcosis in rural population of Moghan plain, Ardebil province of Iran in 2009. | Tropical medicine and international health. 16(Suppl.1):173. | M. Siavashi et al. | 2011 |
| Risk factors for infection with *Echinococcus multilocularis* in Alaska. | **The American journal of tropical medicine and hygiene. 38(2):380–5.** | **J.K. Stehr-Green et al.** | **1988** |
| Echinococcosis in Tibetan populations, western Sichuan Province, China. | **Emerging infectious diseases. 11(12):1866–73.** | **L. Tiaoying et al.** | **2005** |
| Investigation of risk factors for development of human hydatidosis among households raising livestock in Tibetan areas of western Sichuan province. | **Chinese journal of parasitology and parasitic diseases. 19(2):93–96.** | **Q. Wang et al.** | **2001** |
| Fenced pasture: a possible risk factor for human alveolar echinococcosis in Tibetan pastoralist communities of Sichuan, China. | **Acta tropica. 90(3):285–93.** | **Q. Wang et al.** | **2004** |
| Socioeconomic and behaviour risk factors of human alveolar echinococcosis in Tibetan communities in Sichuan, People’s Republic of China. | **The American Journal of Tropical Medicine and Hygiene. 74(5):856–62.** | **Q. Wang et al.** | **2006** |
| Community surveys and risk factor analysis of human alveolar and cystic echinococcosis in Ningxia Hui Autonomous Region, China. | **Bulletin of the World Health Organisation. 84(9):714–21.** | **Y.R. Yang et al.** | **2006** |

**Legend**. In **bold,** studies used for meta-analysis
