# Supplementary material for: Potential risk factors associated with human alveolar echinococcosis: Systematic review and meta-analysis
Source: PLoS Negl Trop Dis. 2017 Jul 17;11(7):e0005801. doi: 10.1371/journal.pntd.0005801 (PMC5531747; doi:10.1371/journal.pntd.0005801)
Supplement: S1 Flow Diagram — (DOC) [file pntd.0005801.s004.doc]

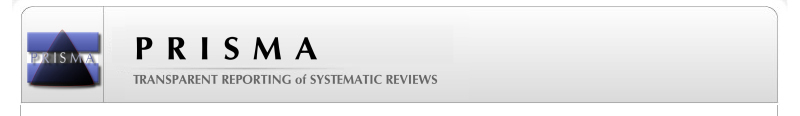
**PRISMA 2009 Flow Diagram**

**Screening**

**Included**

**Eligibility**

**Identification**

Records identified through database searching
(n = 1,009)

Additional records identified through other sources
(n = 0)

Records after duplicates removed
(n = 494)

Records screened
(n = 78)

Records excluded
(n = 416)

Full-text articles assessed for eligibility
(n = 78)

Full-text articles excluded, with reasons
(n = 55)

Studies included in qualitative synthesis
(n = 23)

Studies included in quantitative synthesis (meta-analysis)
(n = 17)
