# Supplementary material for: Potential risk factors associated with human alveolar echinococcosis: Systematic review and meta-analysis
Source: PLoS Negl Trop Dis. 2017 Jul 17;11(7):e0005801. doi: 10.1371/journal.pntd.0005801 (PMC5531747; doi:10.1371/journal.pntd.0005801)
Supplement: S1 Supplementary information — (DOCX) [file pntd.0005801.s005.docx]

CASE-CONTROL studies: Forest and funnel plot analysis on single potential risk factors.

Dog ownership


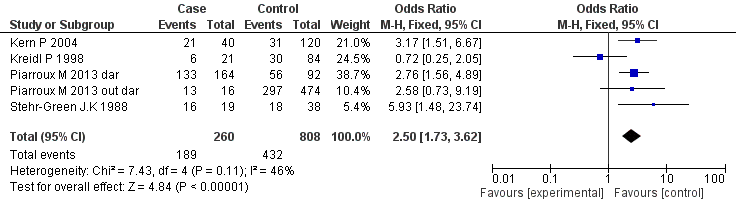


Figure 1.1a. Forest plot on the potential risk factor ’dog ownership’.


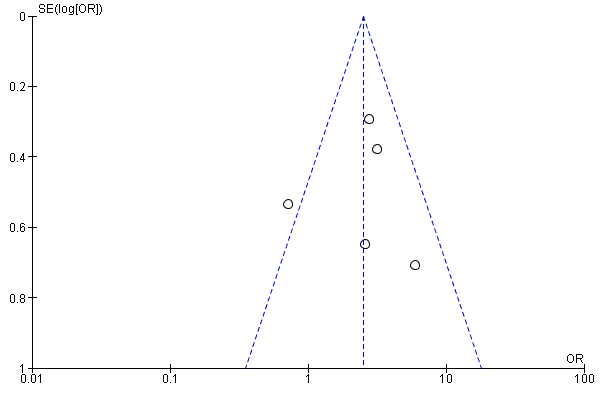


Figure 1.1b. Funnel plot on the potential risk factor ’dog ownership’.

Allowed dog into house


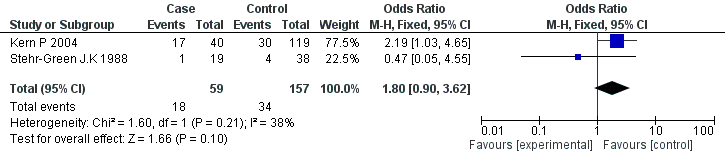


Figure 1.2a. Forest plot on the potential risk factor ’Allowed dog into house’.


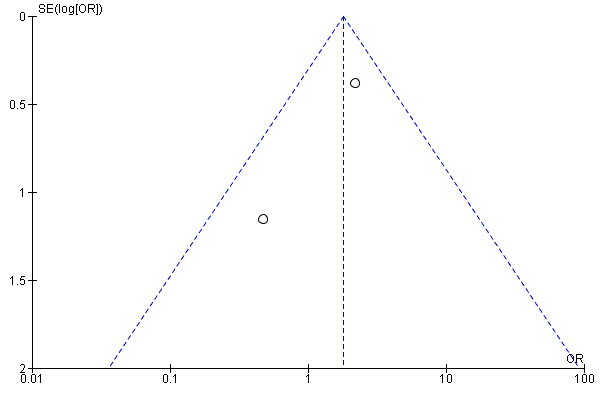


Figure 1.2b. Funnel plot on the potential risk factor ‘Allowed dog into house’.

Play with dogs


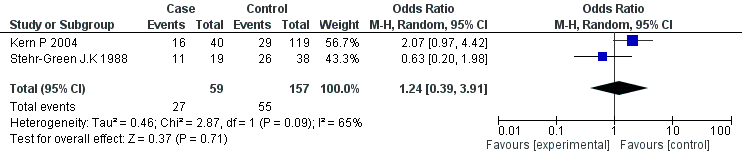


Figure 1.3a. Forest plot on the potential risk factor ’Play with dogs’.


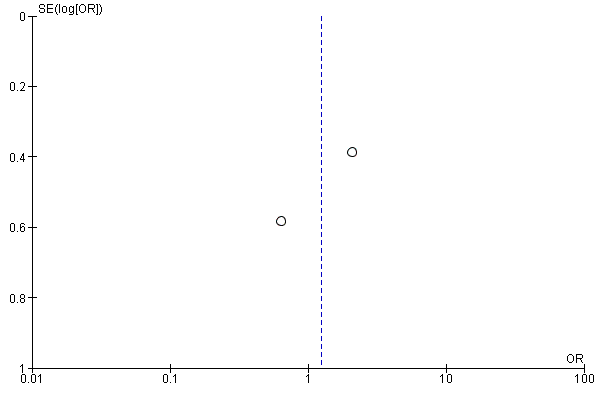


Figure 1.3b. Funnel plot on the potential risk factor ’Play with dogs’.

Cat ownership


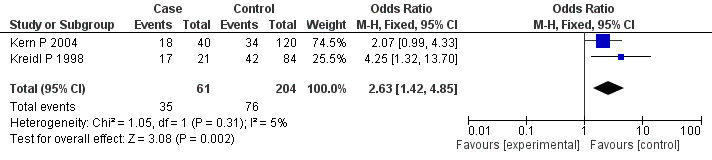


Figure 1.4a. Forest plot on the potential risk factor ’Cat ownership’.


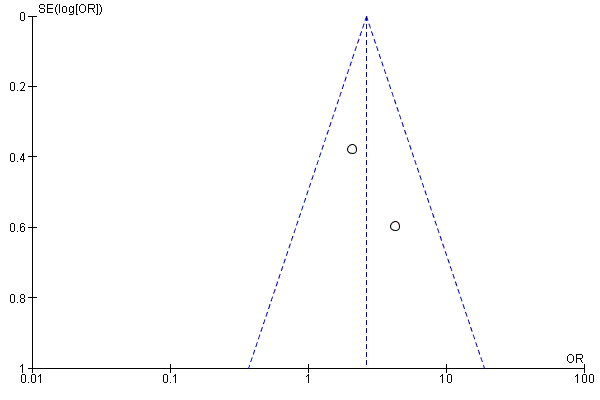


Figure 1.4b. Funnel plot on the potential risk factor ’Cat ownership’.

Living in a rural area


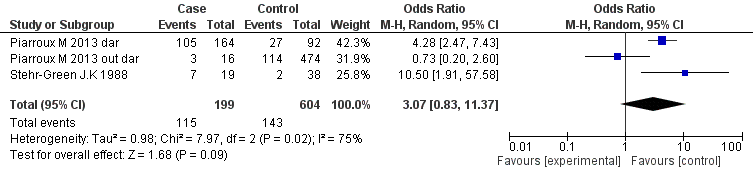


Figure 1.5a. Forest plot on the potential risk factor ‘Living in a rural area’.


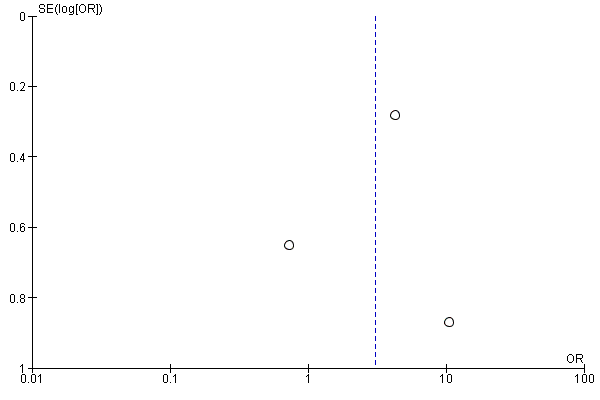


Figure 1.5b. Funnel plot on the potential risk factor ‘Living in a rural area’.

Having a kitchen garden


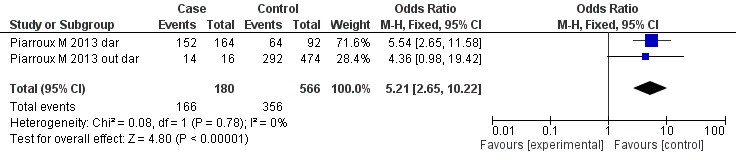


Figure 1.6a. Forest plot on the potential risk factor ‘Having a kitchen garden’.


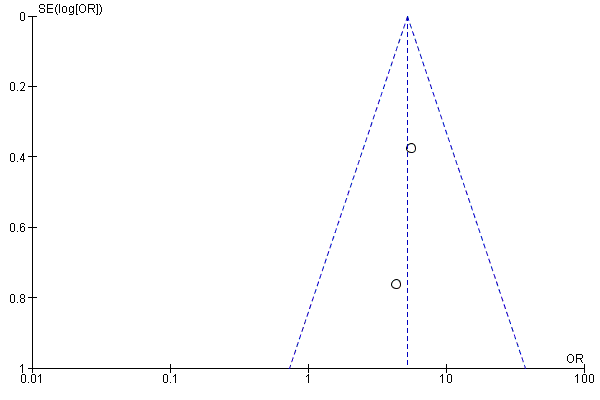


Figure 1.6b. Funnel plot on the potential risk factor ‘Having a kitchen garden’.

Occupation: Farmer


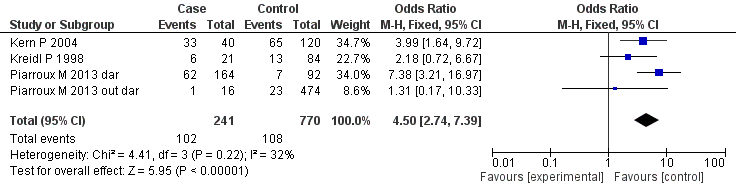


Figure 1.7a. Forest plot on the potential risk factor ’Occupation: Farmer’.


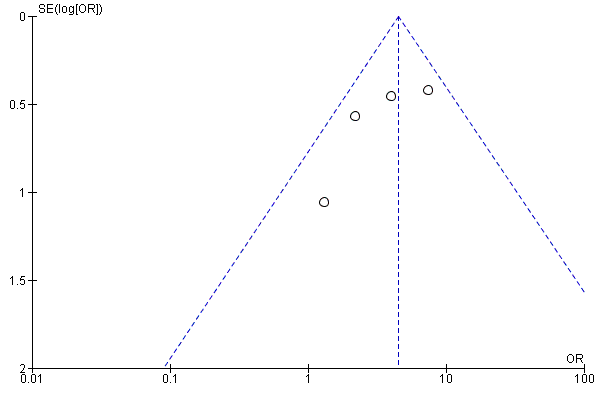


Figure 1.7b. Funnel plot on the potential risk factor ’Occupation: Farmer’.

Haymaking on meadows not adjacent to water


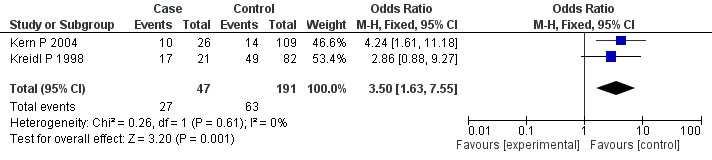


Figure 1.8a. Forest plot on the potential risk factor ‘Haymaking on meadows not adjacent to water’.


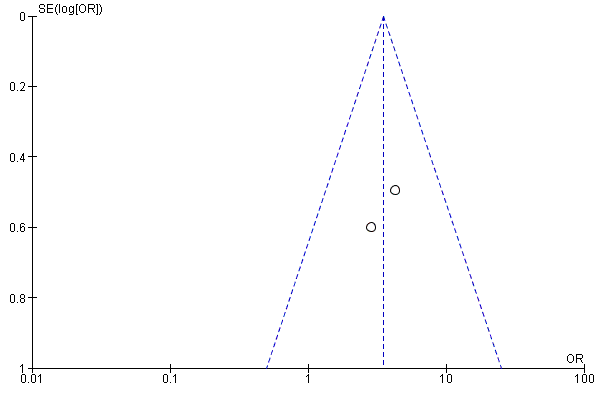


Figure 1.8b. Funnel plot on the potential risk factor ‘Haymaking on meadows not adjacent to water’.

Went to forest for vocational reasons


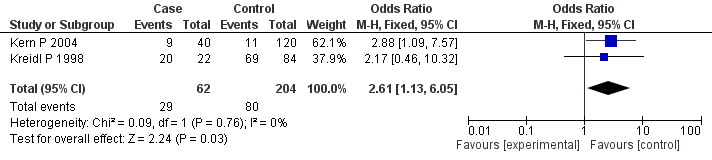


Figure 1.9a. Forest plot on the potential risk factor ‘Went to forest for vocational reasons’.


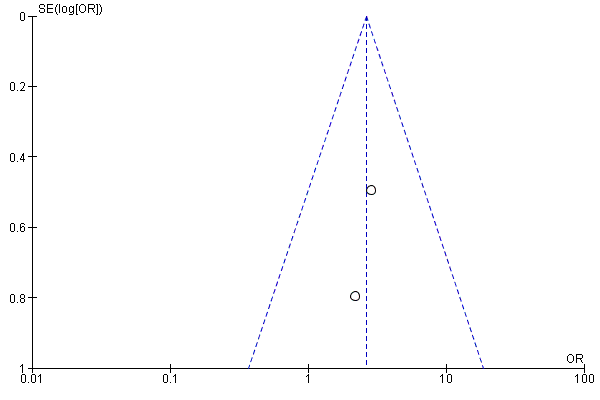


Figure 1.9b. Funnel plot on the potential risk factor ‘Went to forest for vocational reasons’.

Ate unwashed strawberries


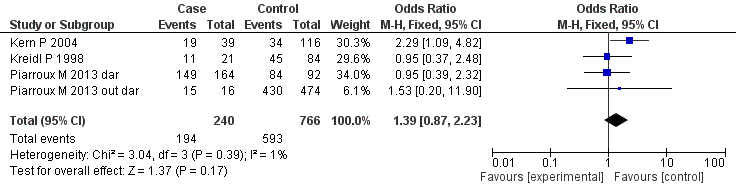


Figure 1.10a. Forest plot on the potential risk factor ‘Ate unwashed strawberries’.


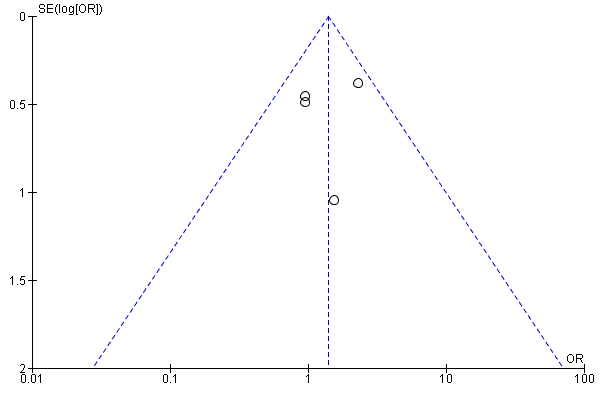


Figure 1.10b. Funnel plot on the potential risk factor ‘Ate unwashed strawberries’.

Chewed grass


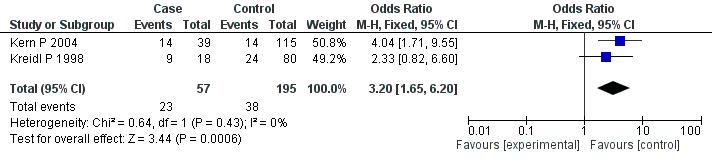


Figure 1.11a. Forest plot on the potential risk factor ‘Chewed grass’.


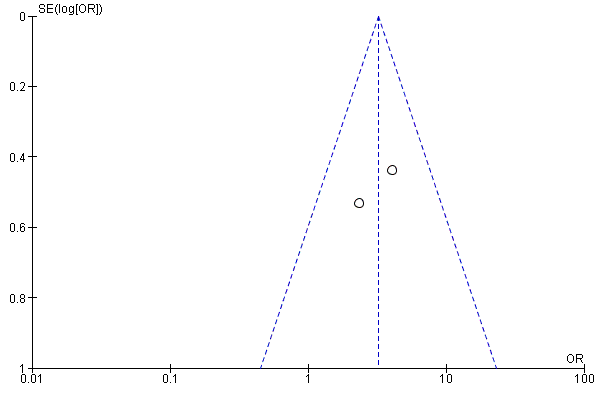


Figure 1.11b. Funnel plot on the potential risk factor ‘Chewed grass’.

Hunting


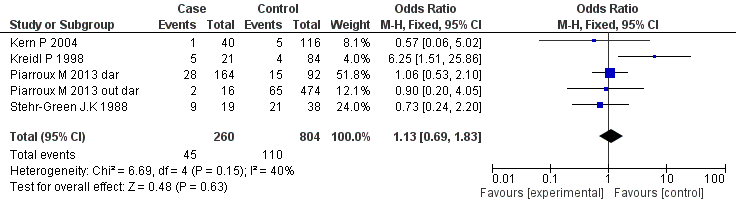


Figure 1.12a. Forest plot on the potential risk factor ‘Hunting’.


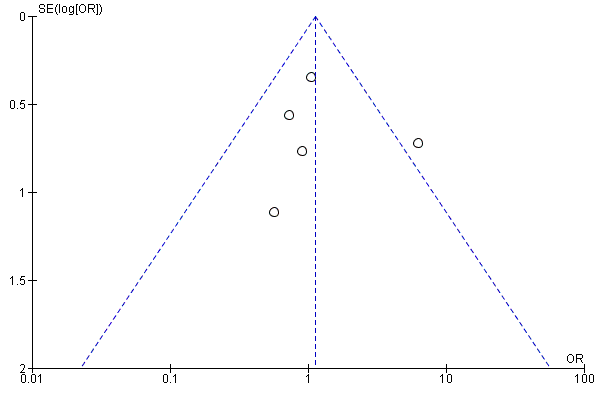


Figure 1.12b. Funnel plot on the potential risk factor ‘Hunting’.

Handled foxes


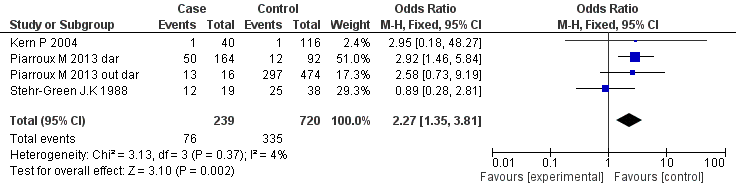


Figure 1.13a. Forest plot on the potential risk factor ‘Handled foxes’.


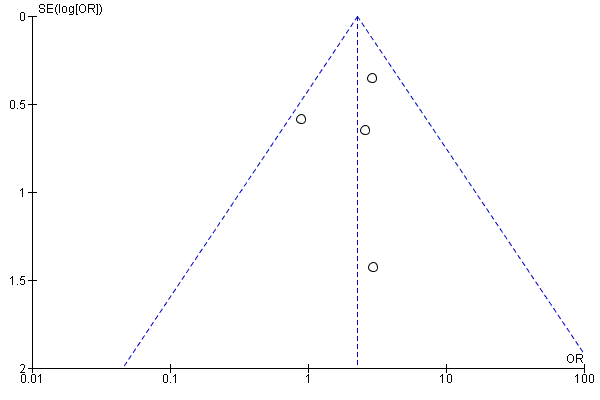


Figure 1.13b. Funnel plot on the potential risk factor ‘Handled foxes’.

Ate mushrooms


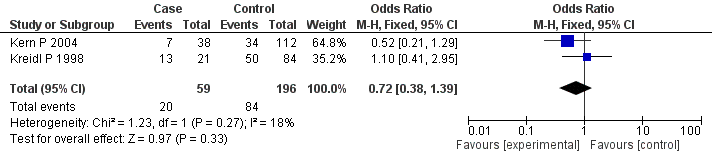


Figure 1.14a. Forest plot on the potential risk factor ‘Ate mushrooms’.


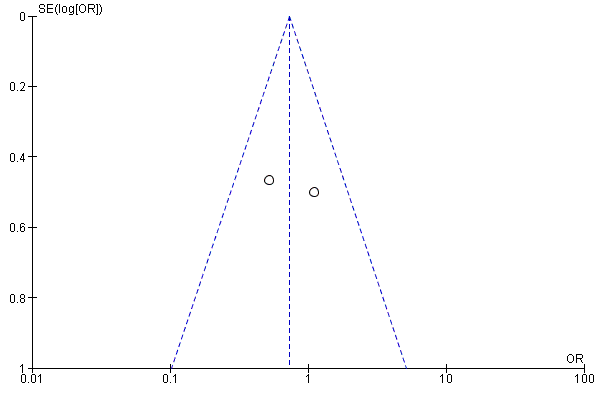


Figure 1.14b. Funnel plot on the potential risk factor ‘Ate mushrooms’.

Ate wild vegetables and fruit


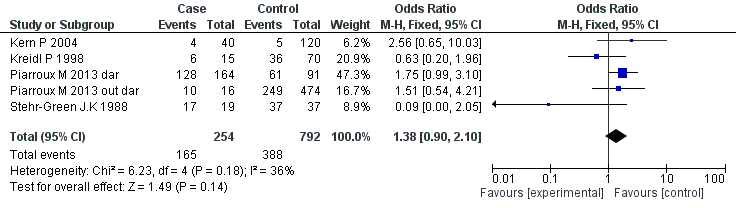


Figure 1.15a. Forest plot on the potential risk factor ‘Ate wild vegetables and fruit’.


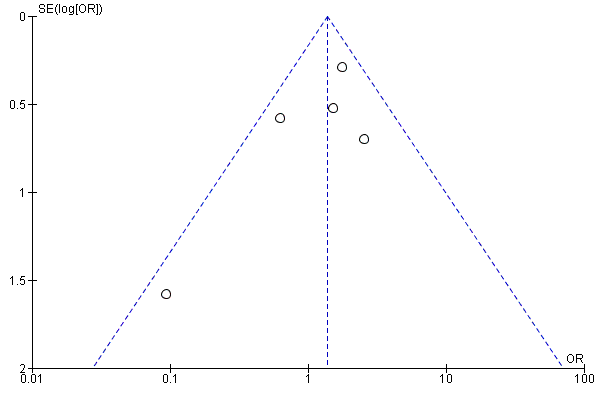


Figure 1.15b. Funnel plot on the potential risk factor ‘Ate wild vegetables and fruit’.

Human leukocyte antigen, HLA


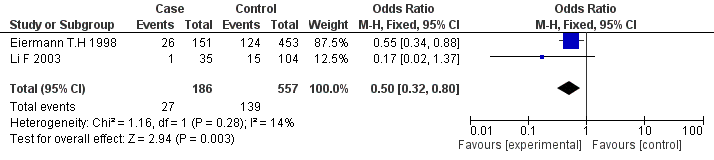


Figure 1.16a. Forest plot on the potential protective factor ‘Human leukocyte antigen, HLA’.


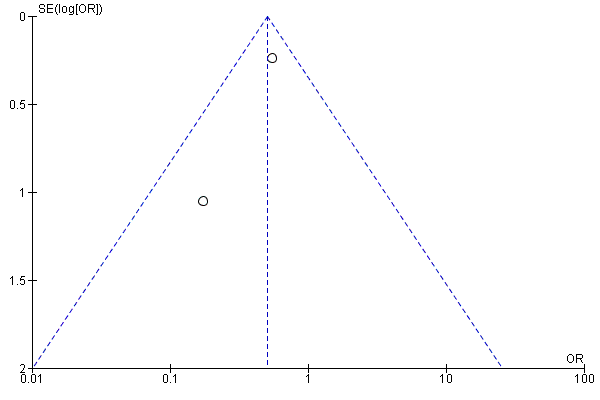


Figure 1.16b. Funnel plot on the potential protective factor ‘Human leukocyte antigen, HLA’.

All forest and funnel plots were prepared using RevMan 5.2 software.
