# Supplementary material for: Potential risk factors associated with human alveolar echinococcosis: Systematic review and meta-analysis
Source: PLoS Negl Trop Dis. 2017 Jul 17;11(7):e0005801. doi: 10.1371/journal.pntd.0005801 (PMC5531747; doi:10.1371/journal.pntd.0005801)
Supplement: S2 Supplementary information — (DOCX) [file pntd.0005801.s006.docx]

CROSS-SECTIONAL studies: Forest and funnel plot analysis on single potential risk factors.

Dog ownership


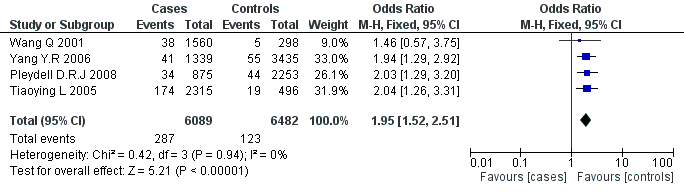


Figure 2.1a. Forest plot on the potential risk factor ‘Dog ownership’.


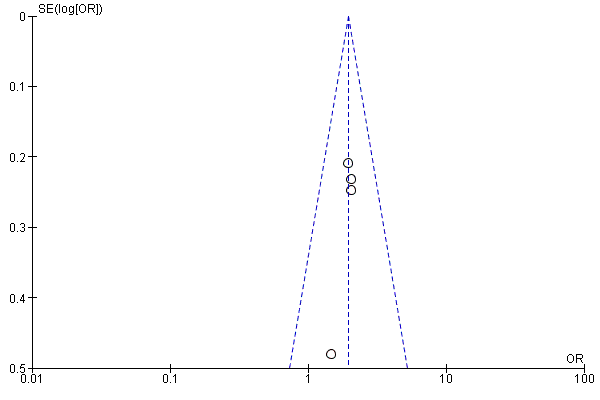


Figure 2.1b. Funnel plot on the potential risk factor ‘Dog ownership’.

Play with dogs


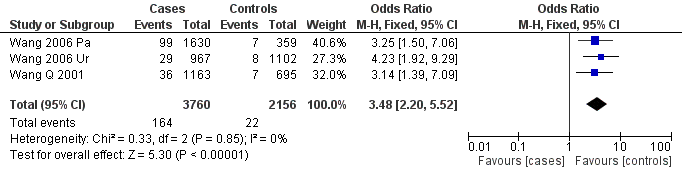


Figure 2.2a. Forest plot on the potential risk factor ‘Play with dogs’.


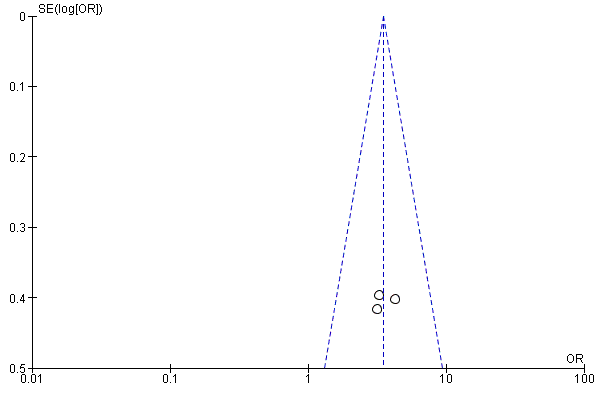


Figure 2.2b. Funnel plot on the potential risk factor ‘Play with dogs’.

Hand wash before eating


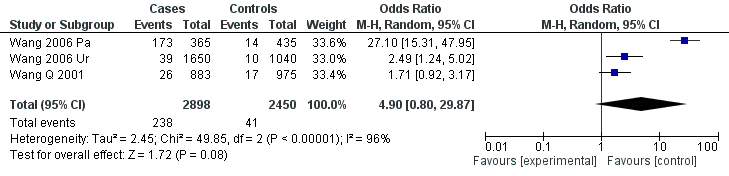


Figure 2.3a. Forest plot on the potential risk factor ‘Hand wash before eating’.


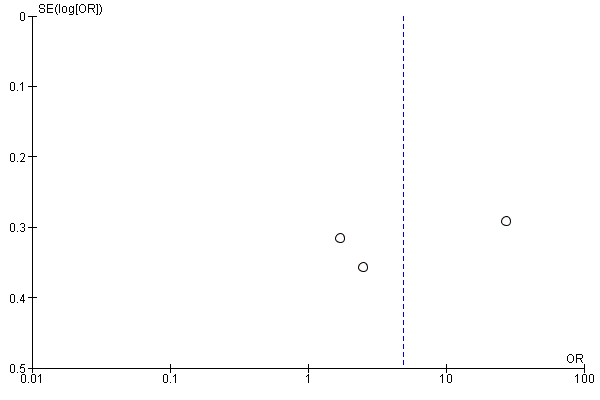


Figure 2.3b. Funnel plot on the potential risk factor ‘Hand wash before eating’.

Gender: female


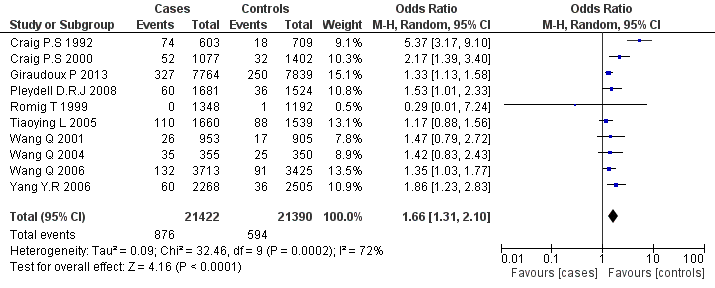


Figure 2.4a. Forest plot on the potential risk factor ‘Gender: female’.


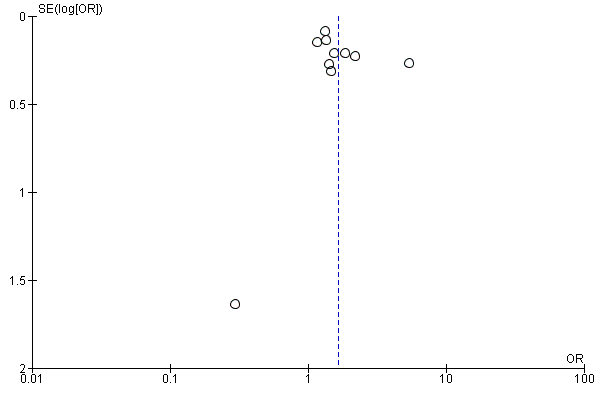


Figure 2.4b. Funnel plot on the potential risk factor ‘Gender: female’.

Age over 20 years


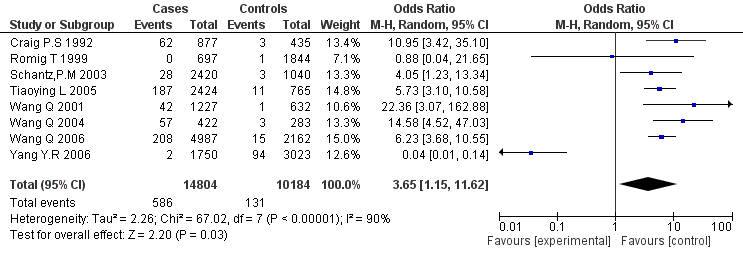


Figure 2.5a. Forest plot on the potential risk factor ‘Age over 20 years’.


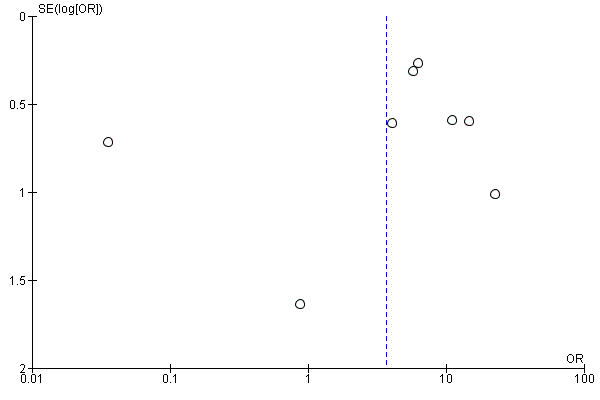


Figure 2.5b. Funnel plot on the potential risk factor ‘Age over 20 years’.

Ethnic group: Tibetan


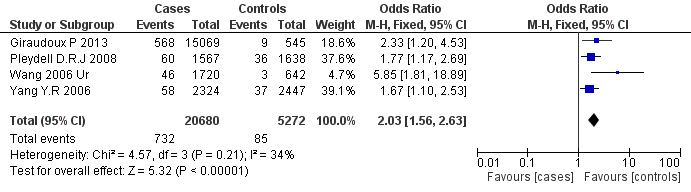


Figure 2.6a. Forest plot on the potential risk factor ‘Ethnic group: Tibetan’.


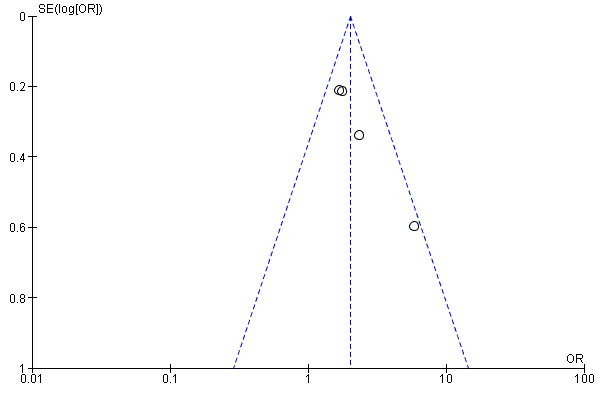


Figure 2.6b. Funnel plot on the potential risk factor ‘Ethnic group: Tibetan’.

Low income


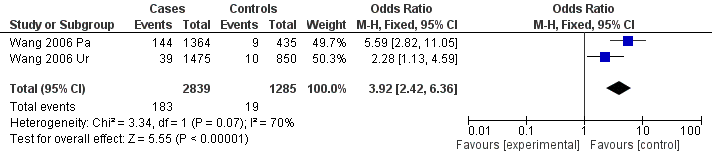


Figure 2.7a. Forest plot on the potential risk factor ‘Low income’.


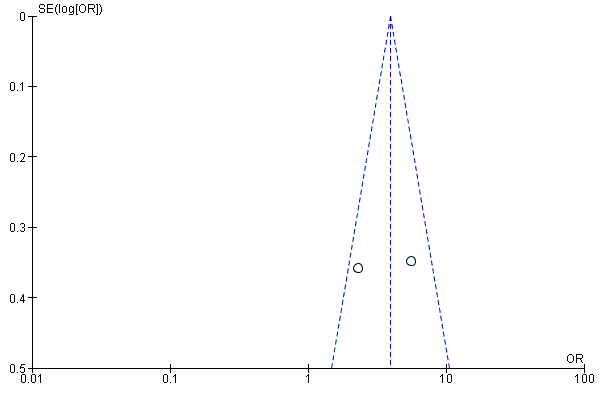


Figure 2.7b. Funnel plot on the potential risk factor ‘Low income’.

Source of drinking water other than well or tap


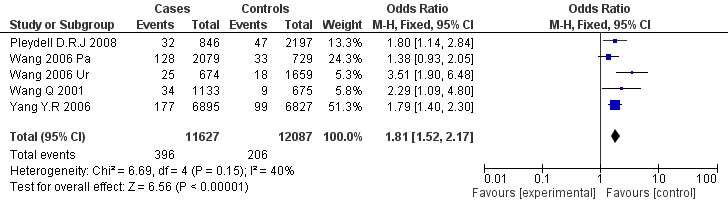


Figure 2.8a. Forest plot on the potential risk factor ‘Source of drinking water other than well or tap’.


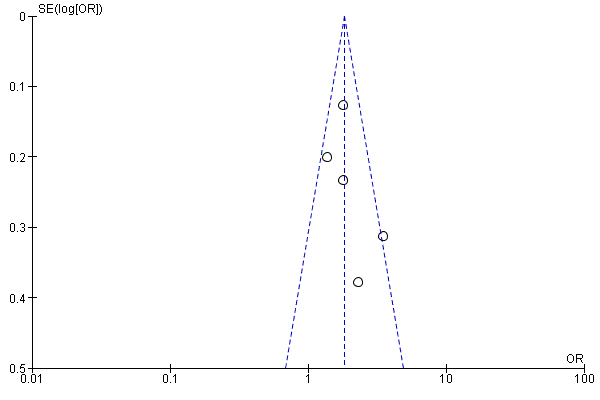


Figure 2.8b. Funnel plot on the potential risk factor ‘Source of drinking water other than well or tap’.

Occupation: farmer


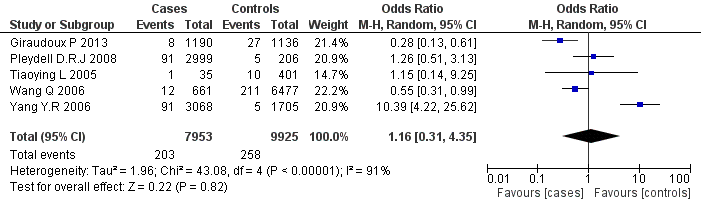


Figure 2.9a. Forest plot on the potential risk factor ‘Occupation: farmer’.


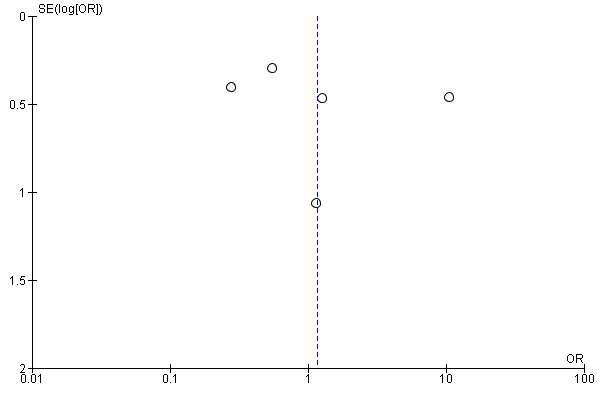


Figure 2.9b. Funnel plot on the potential risk factor ‘Occupation: farmer’.

Occupation: herding


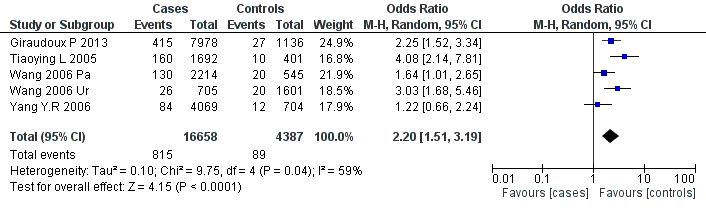


Figure 2.10a. Forest plot on the potential risk factor ‘Occupation: herding’.


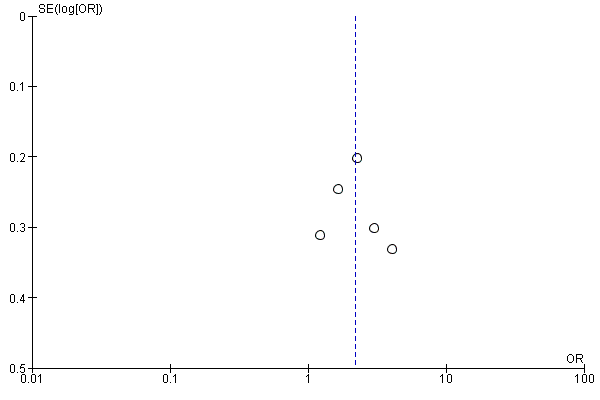


Figure 2.10b. Funnel plot on the potential risk factor ‘Occupation: herding’.

Drinking non-boiled water


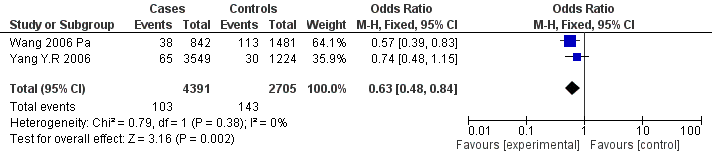


Figure 2.11a. Forest plot on the potential protective factor ‘Drinking non-boiled water’.


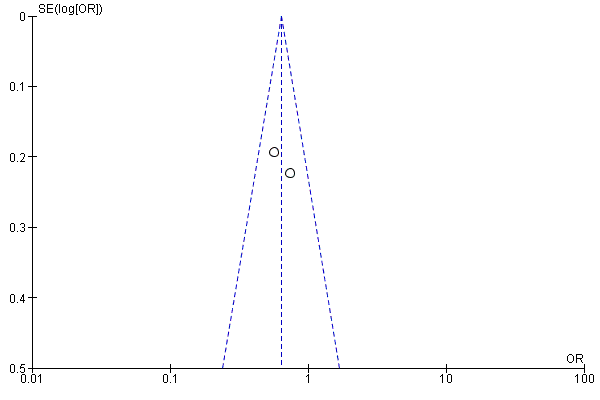


Figure 2.11b. Funnel plot on the protective risk factor ‘Drinking non-boiled water’.

Hunting / handling foxes


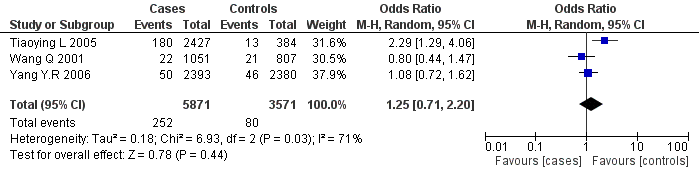


Figure 2.12a. Forest plot on the potential risk factor ‘Hunting / handling foxes’.


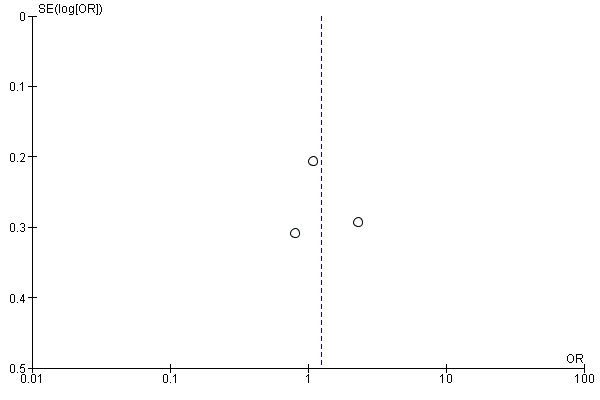


Figure 2.12b. Funnel plot on the potential risk factor ‘Hunting / handling foxes’.

Low education


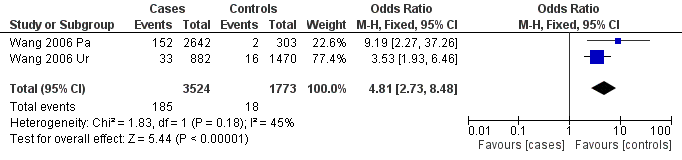


Figure 2.13a. Forest plot on the potential risk factor ‘Low education’.


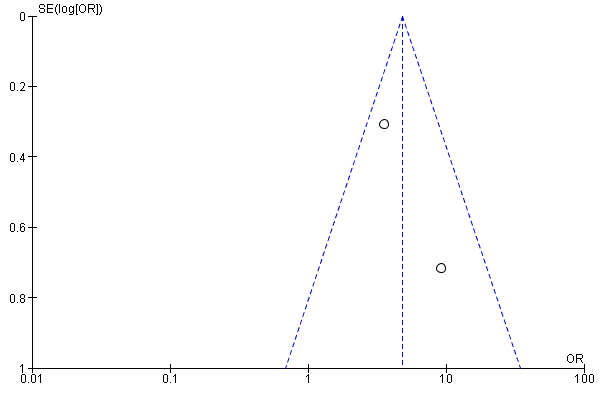


Figure 2.13b. Funnel plot on the potential risk factor ‘Low education’.

All forest and funnel plots were prepared using RevMan 5.2 software.
